# Direct Recognition of *Fusobacterium nucleatum* by the NK Cell Natural Cytotoxicity Receptor NKp46 Aggravates Periodontal Disease

**DOI:** 10.1371/journal.ppat.1002601

**Published:** 2012-03-22

**Authors:** Stella Chaushu, Asaf Wilensky, Chamutal Gur, Lior Shapira, Moran Elboim, Gili Halftek, David Polak, Hagit Achdout, Gilad Bachrach, Ofer Mandelboim

**Affiliations:** 1 Hebrew University-Hadassah School of Dental Medicine, Jerusalem, Israel; 2 Lautenberg Center of General and Tumor Immunology, Hebrew University –Hadassah School of Medicine, IMRIC, Jerusalem, Israel; University of Toronto, Canada

## Abstract

Periodontitis is a common human chronic inflammatory disease that results in the destruction of the tooth attachment apparatus and tooth loss. Although infections with periopathogenic bacteria such as *Porphyromonas gingivalis* (*P. gingivalis*) and *Fusobacterium nucleatum* (*F. nucleatum*) are essential for inducing periodontitis, the nature and magnitude of the disease is determined by the host's immune response. Here, we investigate the role played by the NK killer receptor NKp46 (NCR1 in mice), in the pathogenesis of periodontitis. Using an oral infection periodontitis model we demonstrate that following *F. nucleatum* infection no alveolar bone loss is observed in mice deficient for NCR1 expression, whereas around 20% bone loss is observed in wild type mice and in mice infected with *P. gingivalis*. By using subcutaneous chambers inoculated with *F. nucleatum* we demonstrate that immune cells, including NK cells, rapidly accumulate in the chambers and that this leads to a fast and transient, NCR1-dependant TNF-α secretion. We further show that both the mouse NCR1 and the human NKp46 bind directly to *F. nucleatum* and we demonstrate that this binding is sensitive to heat, to proteinase K and to pronase treatments. Finally, we show *in vitro* that the interaction of NK cells with *F. nucleatum* leads to an NCR1-dependent secretion of TNF-α. Thus, the present study provides the first evidence that NCR1 and NKp46 directly recognize a periodontal pathogen and that this interaction influences the outcome of *F. nucleatum*-mediated periodontitis.

## Author Summary

Periodontal disease is a common bacterial-induced inflammatory process in which *F. nucleatum* and *P. gingivalis* infections lead to the destruction of the teeth supporting attachment apparatus. Previous reports demonstrated that immune cells aggravate the severity of the disease. However, whether NK cells in general and NKp46 (a major killer receptor expressed by NK cells) in particular, play a protective or destructive role in this disease is unknown. Using mice deficient in NCR1 (the mouse orthlogue of NKp46), we demonstrate that oral infection of mice with *F. nucleatum*, but not with *P. gingivalis* results in an NCR1-dependent alveolar bone loss. In addition, we show that *F. nucleatum* is recognized by NCR1 and NKp46 directly and that this recognition leads to the secretion of TNF-α, a central cytokine critically involved in the pathogenesis of periodontal destruction. Collectively, we show that NCR1 and NKp46 play a critical role in the pathogenesis of *F. nucleatum*-mediated periodontitis.

## Introduction

Chronic inflammatory periodontal disease is initiated by several bacterial pathogens. The infection leads to an inflammatory process which results in the destruction of the dental attachment apparatus associated with tooth loss in adults over the age of 35. As such, it affects about one-half of working American adults [1].

The health risks of periodontal disease are not limited to the dentition. Increasing evidence suggests that periodontitis may be an aggravating factor which significantly enhances the risk for developing bacterial endocarditis, aspiration pneumonia, osteomyelitis in children, preterm low birth weight, coronary heart disease, cerebral infarction, atherosclerosis and diabetes mellitus [2]–[7]. Recently, several reports also showed a strong association between rheumatoid arthritis and periodontitis [8]. This correlation may have a causative nature due to protein citrullination by *P. gingivalis* [9]. Nevertheless, although the presence of a periodontal pathogen is prerequisite for periodontitis development, the progression of the disease is dependent on the host innate and adaptive immune responses [10].

NK cells, which are part of the innate immunity, are able to directly kill tumor and virus-infected cells and are also a source for immune mediating cytokines [11]. The function of NK cells is controlled by both inhibitory and activating receptors [12], [13]. When the activity of NK cells is balanced towards activation, it leads to enhanced killing, enhanced production of cytokines, or both [14], [15]. The activating NK receptors includes: the NKp44, NKp30 and NKp46 receptors collectively known as NCR (Natural Cytotoxicity Receptors), 2B4, NKp80, CD16 and NKG2D. The ligands recognized by these receptors are either induced by stress (ligands for NKG2D, see [11], [16]), are viral proteins such as hemagglutinin (ligands for NKp44 and NKp46, see [17], [18]) and pp65 (ligands for NKp30, see [19]), are self-ligands [20] or are unknown tumor ligands. Among the NK activating receptors, the NKp46 receptor (NCR1 in mice) is the only receptor that is expressed specifically by NK cells of both human and mice [11], [21]. The direct *in vivo* role played by NKp46 in the killing of some tumors, influenza virus-infected cells and even self-beta cells was demonstrated using mice in which the NKp46 receptor is “knocked-out” (Ncr1*^gfp/gfp^* mice, see [20], [21]). Importantly, with regard to the current research, NKp46 is not only involved in NK cytotoxicity, but also in production of cytokines [15], [22].

The activity of NK cells in periodontitis has been scarcely studied and to the best of our knowledge, the involvement of the NK killer receptors in the disease was not investigated at all. This is quite surprising since impaired NK cells cytotoxicity has been described in several genetic and acquired conditions associated with periodontal involvement [23]–[26]. It has also been well documented that the local inflammatory response to periodontal bacteria is maintained and amplified by the production of pro-inflammatory cytokines, including TNF-α, IFN-γ and IL1-β [10], [27], [28], the former two being produced by NK cells [29], [30]. In addition, a role for antimicrobial peptides, which are also secreted by NK cells [31], [32], has been also implicated in the pathogenesis of periodontal disease [33], [34].

The present study aimed to address the role of NK cells in general, and of their activating receptor NKp46, in particular, in the pathogenesis of periodontal disease. We demonstrate that *F. nucleatum*, a major etiologic bacteria involved in the pathogenesis of periodontal disease, is directly recognized by the mouse NCR1 and by the human NKp46 receptors and we show that the interaction between NCR1 and *F. nucleatum* probably leads to a rapid and specific secretion of TNF-α, resulting in alveolar bone loss. Collectively our results demonstrate that NK cells through their killing receptor NKp46 play a critical role in the pathogenesis of *F. nucleatum*-mediated periodontal disease.

## Results

### NCR1 controls the *F. nucleatum*-mediated alveolar bone loss

**Figure 1:**
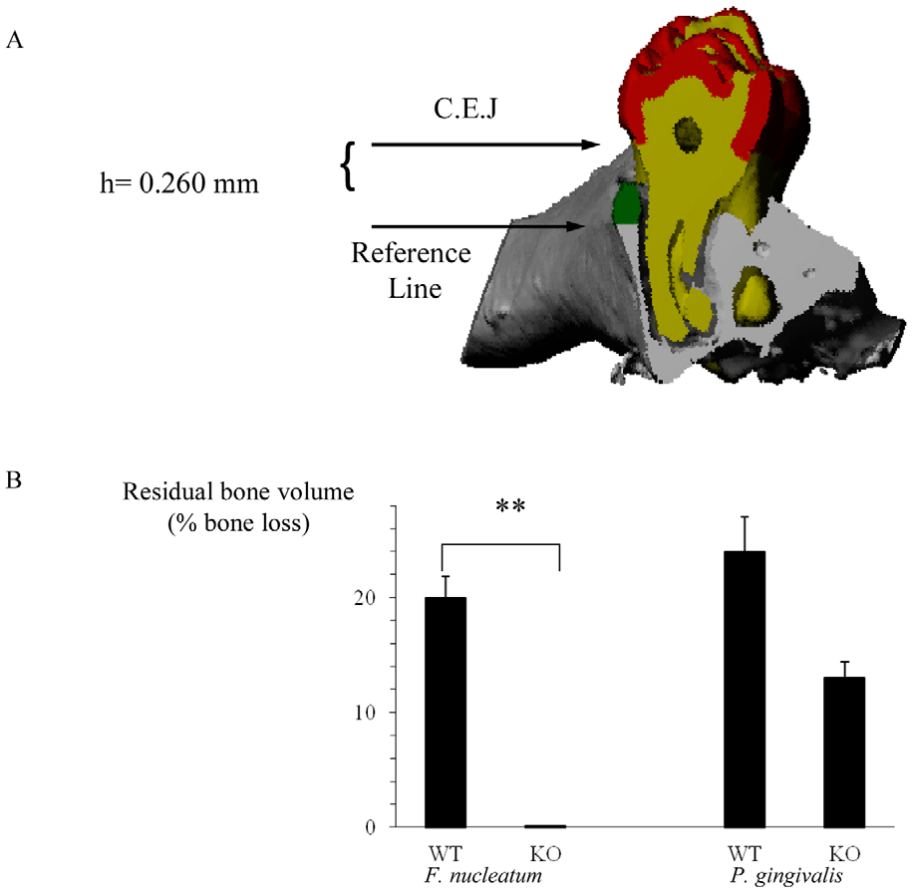
NCR1-dependent bone loss. (A) Mesio-distal view on a random section. Red, yellow, gray and green represent the enamel, dentin, bone and the residual bone area above the reference line, respectively. (B) Ncr1*^+/+^* (WT) and Ncr1*^gfp/gfp^* (KO) mice (n = 8 in each group) were challenged orally three times at 2-day intervals with inoculums of *F. nucleatum* and of *P. gingivalis* (2×10^8^ CFU in 0.2 ml of PBS and 2% carboxymethylcellulose). Six weeks later, the jaws were harvested and the residual alveolar bone volume (mm^3^×10^−3^) was measured. Error bars represent SD. Data represent percentage of bone loss ± SD and are average of two independent experiments. ** *p*<0.01. The difference in bone loss between the KO and the WT mice following *P. gingivalis* inoculation was not statistically significant, *p*<0.09.

The important clinical outcome of oral inflammation that is induced by periodontal pathogens is the degradation of gingival connective tissue and the loss of alveolar bone which supports the teeth. Although the mechanisms underlying the inflammatory process which leads to the destruction of the supporting apparatus are not fully understood, TNF-α was demonstrated to be critical for disease induction [35]–[37]. Because TNF-α is secreted by NK cells and because NKp46 triggering can lead to TNF-α secretion we wondered whether NCR1 is involved in alveolar bone loss. To investigate this, we induced experimental periodontitis in wild type C57BL/6 mice and in the NCR1 knockout mice (Ncr1*^gfp/gfp^*, C57BL/6 background) that were generated in our laboratory by replacing the Ncr1 gene with GFP [21]. Mice were orally infected with *F. nucleatum* and with *P. gingivalis* and the levels of alveolar bone loss were quantified by micro-CT ([38], [39], Figure 1A). Around 20% bone loss was observed in the Ncr1^+/+^ wild type C57BL mice infected with *F. nucleatum*, compared to the Ncr1*^gfp/gfp^* KO mice in which bone loss was not detected at all (Figure 1B). Infection with *P. gingivalis* also resulted in alveolar bone loss which was similar to that of *F. nucleatum*. However, this bone loss was observed in both wild type and Ncr1*^gfp/gfp^* KO mice and no statistically significant differences were observed between the mice (Figure 1B).

### Infiltration of immune cells into infected subcutaneous chambers

The experimental periodontis model is not suitable for investigating NK cell activities because of technical problems, tissue limitations and due to ethical restrictions. Therefore, to further investigate the role played by NK cells in *F. nucleatum* infection we used subcutaneous chambers which were implanted into mice in the dorsolumbar region [40], [41]. This model allows the direct quantification of inflammatory cells and mediators in the inflammatory exudates following bacterial challenge [40].

Subcutaneous chambers were implanted in Ncr1*^gfp/gfp^* KO mice. Two weeks after the implantation, when the outer incision healed completely and the chambers became encapsulated by a thin vascularized layer of fibrous connective tissue, the chambers were inoculated with the two periodontal pathogens *F. nucleatum*, *P. gingivalis*, or with *Escherichia coli* (*E. coli*, used as a non–periodontal gram negative bacterial control).

**Figure 2:**
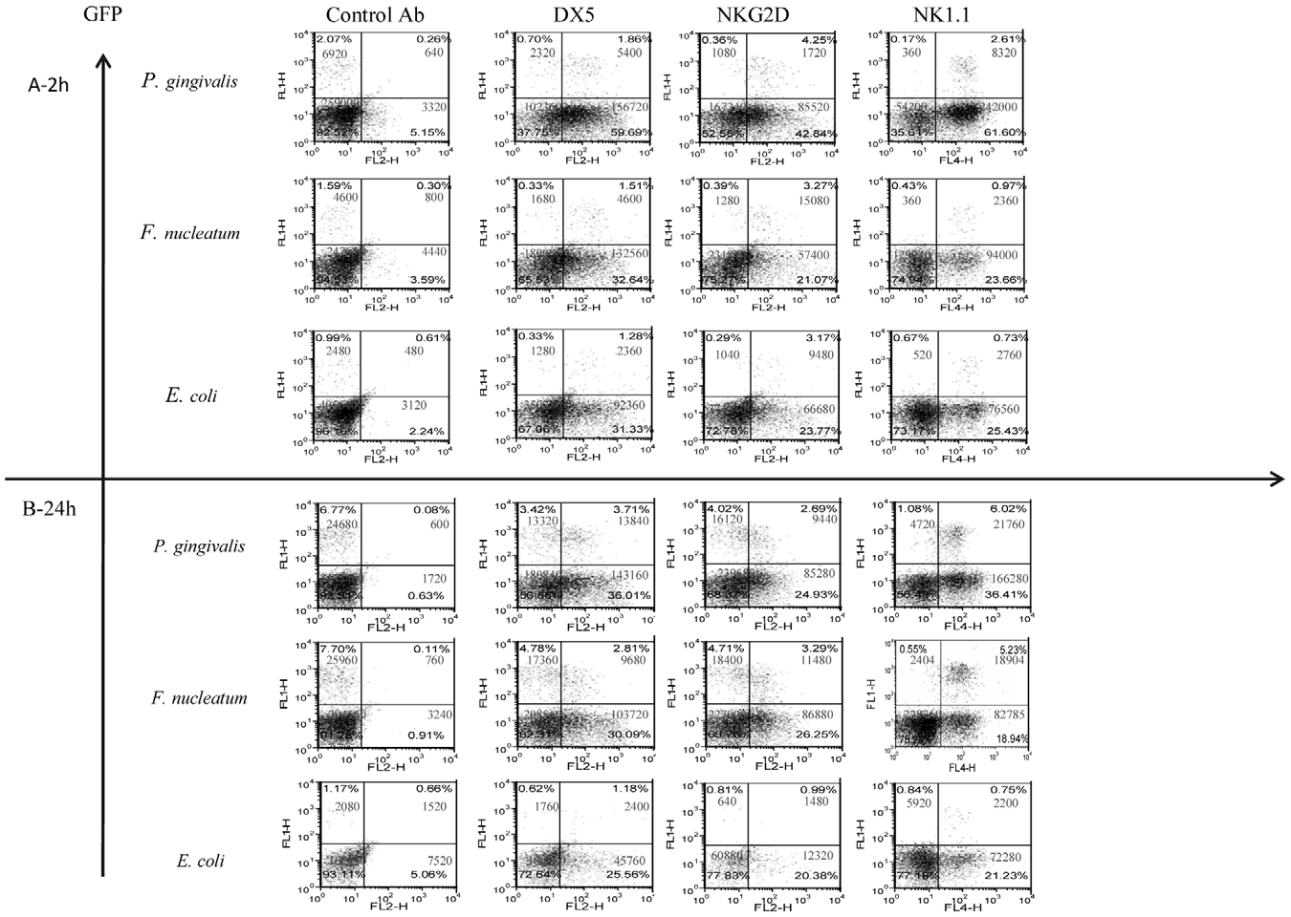
Infiltration of cells into challenged chambers. Immune cells in the chambers were analyzed by FACS 2 hours (A) and 24 hours (B) following bacteria inoculation. The intrinsic GFP labeling marks mainly NK cells. Staining was performed with Phycoerythrin conjugated anti-DX5 and anti-NKG2D mAbs. Staining with NK1.1 was visualized by using Cy-5 conjugated strepavidin. The cell percentage and cell numbers is indicated in each quadrant. One representative experiment out of four is shown.

In agreement with previous reports [41], [42], a rapid and massive leukocyte infiltrate was observed in the chamber exudates following bacterial inoculation (Figure 2). NK cells (GFP-positive) were detected in the chambers as early as 2 hours post inoculation (Figure 2, left quadrates). The percentages and the numbers of NK cells increased at 24 hours following the inoculation, except for the *E. coli*-inoculated group (Figure 2).

To further characterize the NK population that accumulated in the chambers following bacteria inoculation, we stained the cell content of the chambers with antibodies directed against NK cell receptors; NKG2D, DX5 and NK1.1. As can be seen in Figure 2, all of the GFP labeled NK cells were NK1.1 positive and CD3 negative (not shown) and most of them, at 2 hours post infection, were DX5 and NKG2D positive. A reduction in the expression of DX5 and of NKG2D on NK cells was observed twenty-four hours following the inoculation of *F. nucleatum* and *P. gingivalis* (Figure 2). Notably, NK cells (GFP positive) were the minor immune cell population present in the chambers (Figure 2) as most of cells that express NK cell markers such as NKG2D, DX5 and NK1.1, were actually GFP negative, (Figure 2).

**Figure 3:**
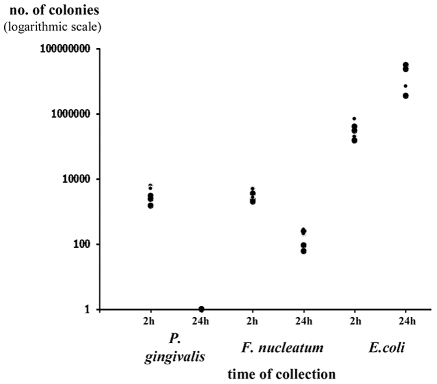
Survival of bacteria in chamber exudates. Viable CFU from chamber exudates were determined, 2 h and 24 h following inoculation of *P. gingivalis*, *F. nucleatum* or *E. coli*. Each symbol represents an individual mouse. One representative experiment out of four is shown. The y axis represents number of colonies (logarithmic scale).

The presence or absence of NKp46 did not affect bacterial loads in the chambers (data not shown and figures below). Furthermore, both *P. gingivalis* and *F. nucleatum* bacteria were detected in the chambers 2 hours following inoculation and little or no bacteria was detected at 24 hours following inoculation (Figure 3 depicting the Ncr1*^gfp/gfp^* mice, and data not shown). In contrast, the *E. coli* load was significantly increased during the tested period (Figure 3).

Thus, we conclude that the vast majority of lymphocytes in the chambers are not NK cells, that the *in vivo* accumulation of lymphocytes in the chambers is rapid, that low percentages and numbers of NK cells are observed in the chambers and that the NK cell percentages and numbers increase with time concomitantly with the disappearance of the two periodontal pathogens.

### *F. nucleatum* inoculation leads to an NCR1-dependent accumulation of TNF-α

**Figure 4:**
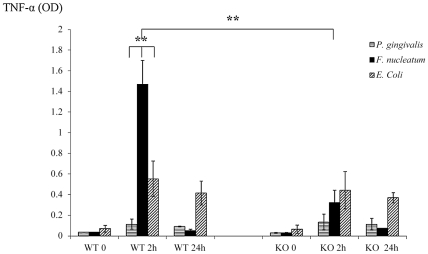
Cytokine profiles in challenged chambers. TNF-α levels were determined by ELISA in chamber exudates of Ncr1*^+/+^* (WT) and Ncr1*^gfp/gfp^* (KO) mice at baseline (0), 2 h and 24 h following bacterial challenge. Data represent absorbance at 650 nm ± SD and are average of three different experiments. ** *p*<0.01.

NKp46/NCR1 is an activating receptor involved in the killing of virus-infected, tumor cells and self-cells [20], [21] and in the secretion of IFN-γ and of TNF-α in response to various stimulations [29]. We therefore next examined whether the absence of NCR1 will affect the cytokines milieu that is present in the chambers exudates. The exudates of all chambers were collected at time 0 (before inoculation), at 2 and at 24 hours post inoculation and IFN-γ and TNF-α levels were measured. Under the tested experimental conditions, the levels of IFN-γ in the chambers could hardly be detected (data not shown). In contrast, a significant and rapid increase in the levels of TNF-α was observed in the Ncr1*^+/+^* WT mice, 2 hours following the challenge with *F. nucleatum* (Figure 4). This elevation was NCR1 dependent, since the levels of TNF-α in the *F. nucleatum*-challenged Ncr1*^gfp/gfp^* KO mice were only modestly increased (Figure 4).

In agreement with the above results, twenty four hours post inoculation of *F. nucleatum*, concomitantly with the disappearance of the bacteria (Figure 3), the TNF-α amounts in the exudates dropped to minimal in both the Ncr1*^+/+^* WT and the Ncr1*^gfp/gfp^* KO mice (Figure 4).

Interestingly, *P. gingivalis* did not induce TNF-α secretion. Increase in TNF-α secretion was also observed following *E. coli* inoculation however this increase was NCR1 independent and was less pronounced as compared with *F. nucleatum*-mediated secretion (Figure 4). Furthermore, in agreement with the presence of *E. coli* in the chambers during the entire tested time period (Figure 3), the increased TNF-α secretion observed following *E. coli* inoculation did not significantly changed between 2 hours and 24 hours.

We also tried performing an intracellular staining for TNF-α in the NK cells isolated from the chambers. However, these NK cells did not survive the 5 hours Brefeldin A treatment, and without this treatment we could not detect TNF-α.

### Immune cell accumulation in the infected chambers in the presence and in the absence of NCR1

To better characterize the immune cell populations present in the inflamed chambers and to investigate whether in the absence of NCR1 the percentage and the number of immune cells present in the chambers will be altered, we implanted chambers in the NCR1 knockout, Ncr1*^gfp/gfp^* mice (KO) and in the heterozygous, Ncr1*^+/gfp^* mice (HET, we used the Ncr1*^+/gfp^* mice because the heterozygous mice behaves similarity to the WT mice, but have an additional advantage as all NK and NK-like cells are GFP-positive, [21]). Since the NCR1 effect was observed 2 hours following *F. nucleatum* injection (see above figures) we inoculated the HET and KO mice with *F. nucleatum* and assayed for the presence of various immune cells two hours post infection.

**Figure 5:**
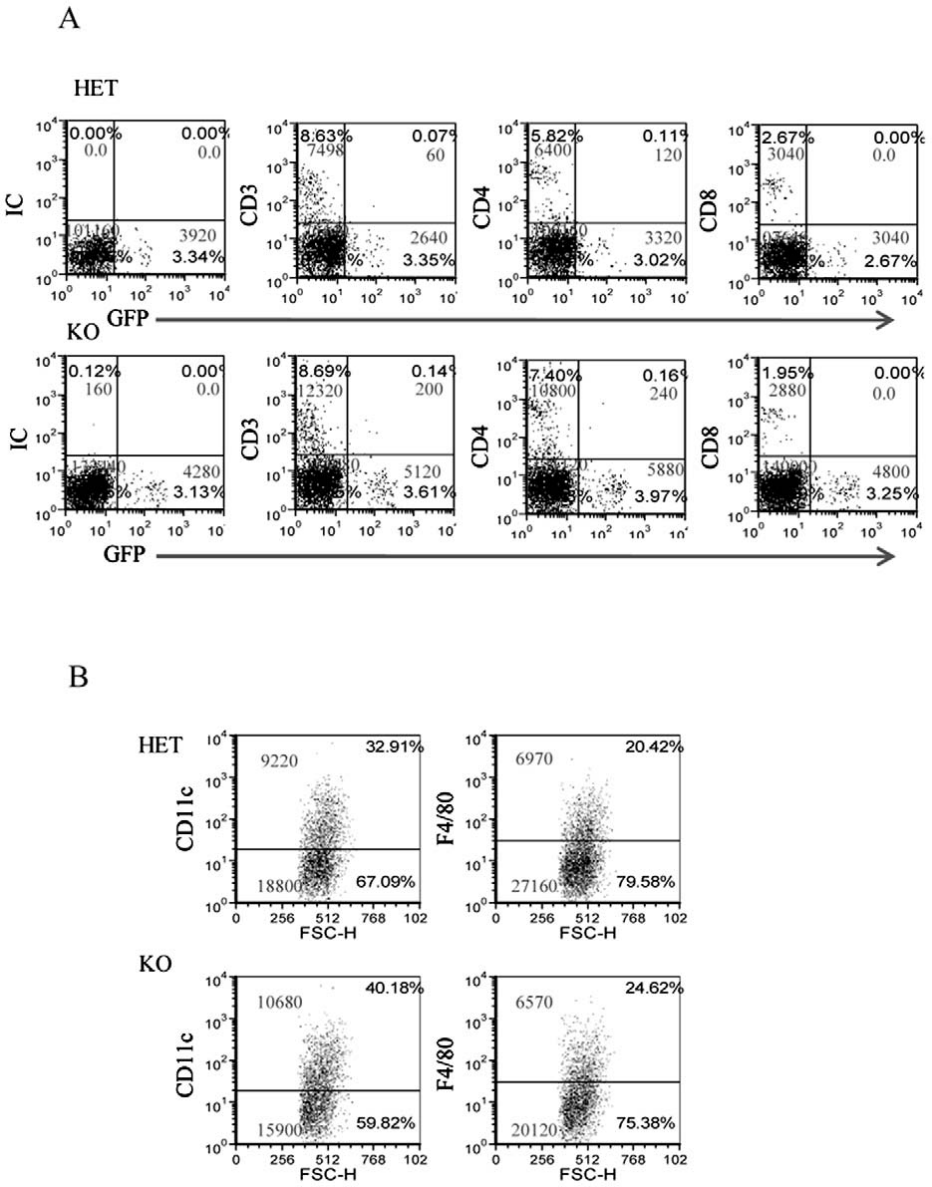
The infiltration of immune cells into the *F. nucleatum* challenged chambers is NCR1-independent. The presence of immune cells; (A) lymphocytes and (B) DC (identified by CD11c) and macrophages (identified by F4/80) in the chambers were analyzed by FACS, 2 hours following the injection of *F. nucleatum*. The mAb used for staining is indicated in the Y axis. IC is isotype control. The percentage of the various cells and the cell numbers are indicated in each quadrant. One representative experiment out of four is shown.

Surprisingly, as can be seen in Figure 5, more lymphocytes had accumulated in the *F. nucleatum* infected chambers in the absence of NCR1 (the reasons for this are currently unknown). The T cell numbers (CD3+) were, in general, twice as large as the NK cell numbers and most of these T cells were helper CD4+ cells (Figure 5A). Macrophages (detected by F4/80+ staining) and DC (detected by CD11c staining) were also present in the chambers more or less at equal levels, irrespectively of whether NCR1 is present or is absent (Figure 5B).

### NCR1 and NKp46 directly interact with *F. nucleatum*

**Figure 6:**
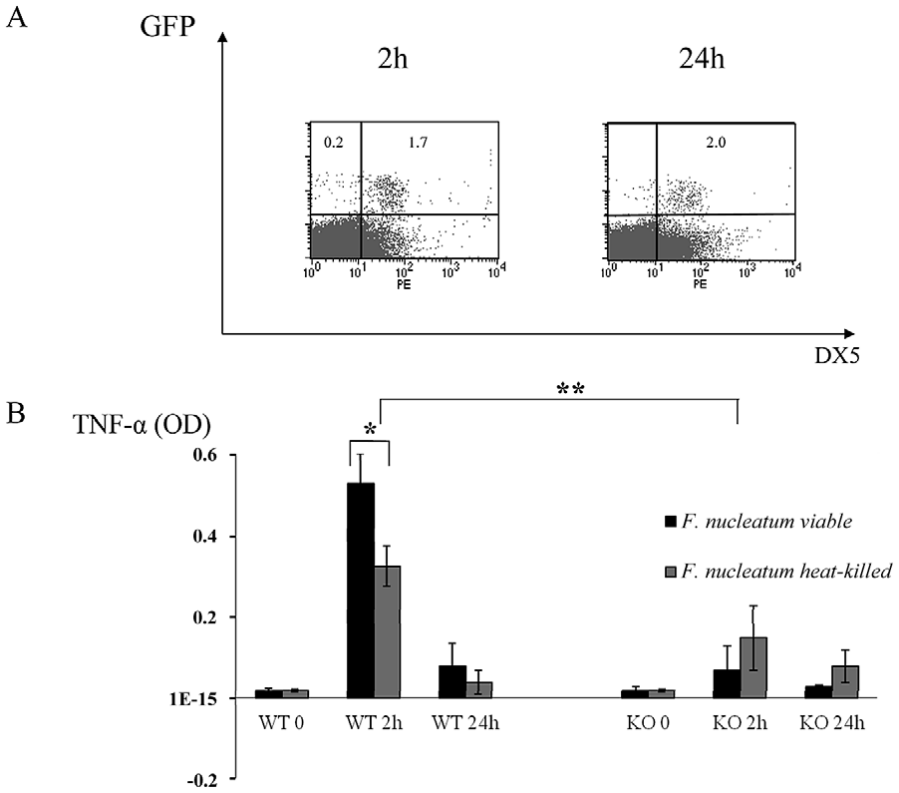
*In vivo* response to heat-treated *F. nucleatum* bacteria. (A) FACS analysis of chamber exudates, 2 hours and 24 hours following challenge with heat-killed *F. nucleatum*. Staining was performed with Phycoerythrin conjugated anti-DX5 mAb. The percentages of GFP^+^ NK cells are indicated. One representative experiment out of three is shown. (B) TNF-α level in chamber exudates of Ncr1*^+/+^* (WT) and Ncr1*^gfp/gfp^* (KO) was determined by ELISA, 2 and 24 hours following challenge with viable and heat-killed *F. nucleatum*. Data represent absorbance at 650 nm ± SD and are average of two different experiments. * *p*<0.05, ** *p*<0.01.

To test whether live bacteria is required for triggering the NCR1-mediated TNF-α secretion we repeated the *in vivo* chamber model experiments with heat-treated *F. nucleatum*. As seen in Figure 6A, 2 hours after inoculation with heat-killed *F. nucleatum*, the percentages of NK cells in the chambers were similar to those observed with the untreated, viable *F. nucleatum* (compare Figures 2 and 6). In contrast, 24 hours post inoculation, the percentages of NK cells in the chambers inoculated with the heat-killed *F. nucleatum* were not elevated (as observed with the untreated *F. nucleatum*, Figure 2), but rather stayed constant (Figure 6A).

We next tested whether the TNF-α production will be affected by heat treatment. As can be seen in Figure 6B, a significant secretion of TNF-α was still detected following the inoculation of a heat-killed *F. nucleatum* bacteria (compared with the KO mice), however this TNF-α secretion was significantly less pronounced as compared with the *F. nucleatum* live bacteria. Thus, we concluded that heat treatment of the bacteria affects NK cell accumulation in the chamber and that it also slightly affects the NCR1-dependent TNFα secretion.

**Figure 7:**
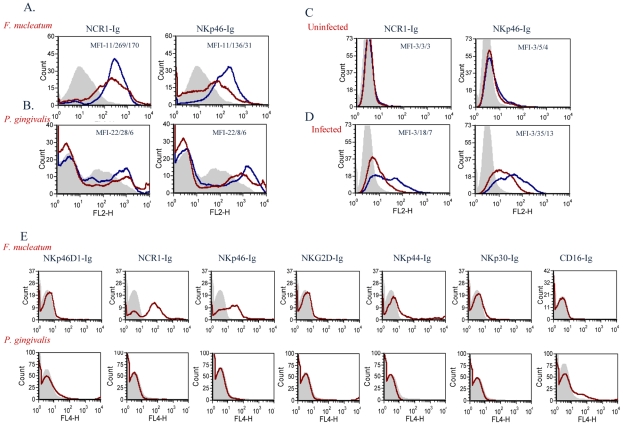
Direct binding of NCR1 and NKp46 to *F. nucleatum*. *F. nucleatum* (A), *P. gingivalis* (B), uninfected 721.221 cells (C), and 721.221 cells infected with influenza PR8 virus (D), were stained with NCR1-Ig (left) and with NKp46-Ig (right) at two different dosages; 5 µg, blue line and 1 µg, red line. Staining was visualized using a Phycoerythrin conjugated anti-human Ig antibody. The filled grey histograms represent control staining with the NKp46D1-Ig fusion protein. The Median Fluorescence Intensity (MFI) values of the D1-Ig, NCR1-Ig and NKp46 staining are indicated in each of the histograms. One representative experiment out of five is shown. (E) *F. nucleatum* (upper) and *P. gingivalis* (lower) bacteria were stained with various activating NK cell receptors fused to Ig (indicated above the quadrants). Figure shows one representative experiment out of three performed.

To test whether the mouse NCR1 and the human NKp46 directly interact with *F. nucleatum* we used fusion proteins (generated as previously described [17]), in which the extracellular domains of NCR1 and of NKp46 are fused to the Fc portion of human IgG1. As a negative control we used an Ig fusion protein made of the membrane proximal domain of NKp46 (named D1), that does not include the NKp46 site required for its binding [17], [21], [43]. Importantly, as can be seen in Figure 7A, a specific, dose dependent, binding of NCR1-Ig and NKp46-Ig was observed to *F. nucleatum*, whereas little or no binding was observed to *P. gingivalis* (Figure 7B).

We have previously showed that NKp46 and NCR1 recognize influenza virus hemagglutinins [17], [21], [43]. To compare the efficiency of the interactions of NKp46 and NCR1 with *F. nucleatum* to that of hemagglutinin, we infected 721.221 cells with the PR8 influenza virus. As can be seen in Figure 7C, in the absence of infection little or no binding was observed when either NKp46-Ig or NCR1-Ig were used. In agreement with previous reports [17], [21], [43], following influenza infection, the binding of NKp46-Ig and NCR1-Ig was substantially increased (Figure 7D). The increased binding was dose-dependent (Figure 7) and was noticed with similar concentrations of fusion proteins that were used to detect binding to *F. nucleatum*.

For unknown reasons the *P. gingivalis* staining is sometimes observed as a smeared peak (Figure 7B) and sometimes appeared as a single peak (Figure 7E). Thus, to further demonstrate that the *F. nucleatum* recognition by NKp46/NCR1 is specific and that *P. gingivalis* is indeed not recognized by NKp46/NCR1 we stained the *P. gingivalis* and the *F. nucleatum* bacteria with various killer receptors fused to Ig. As can be seen in Figure 7E little or no staining of the Ig-fusion proteins (NKp46D1-Ig, NCR1-Ig, NKp46-Ig, NKG2D-Ig, NKp44-Ig, NKp30-Ig and CD16-Ig) was observed with the *P. gingivalis*, whereas *F. nucleatum* was recognized only by the human NKp46-Ig and its mouse orthologue NCR1-Ig (Figure 7E).

### Binding of NCR1 and NKp46 to *F. nucleatum* is heat, proteinase K and pronase sensitive

We have previously shown that the recognition of influenza hemagglutinin by NCR1 and by NKp46 requires the sialylation of these receptors and that it is sensitive to digestion with neuroaminidase (NA) [17], [21], [43]. We therefore tested whether binding NCR1 and NKp46 to the unknown *F. nucleatum* ligand also involves sialic acid residues. For this, we treated the proteins with bacterial neuraminidase and observed that such treatment did not affect the binding to *F. nucleatum* (data not shown), suggesting that binding of NCR1 and NKp46 to the fusobacterial ligand is mechanistically different from the binding to viral hemagglutinins.

To further delineate the nature of the fusobacterial ligand, we tested the binding of NCR1-Ig and NKp46-Ig to heat-killed *F. nucleatum*, or to *F. nucleatum* that was treated with enzymes (proteinase K or pronase) known to modify proteins on bacterial surfaces. Because the proteinase K or pronase enzymes are active at 55°C we also tested whether NCR1-Ig and NKp46-Ig interact with *F. nucleatum* after heating the bacteria at 55°C for 1 hour and observed efficient binding (data not shown). Furthermore, as shown in Supplementary Figure S1, all treatments did not affect the integrity or the presence of the bacteria.

**Figure 8:**
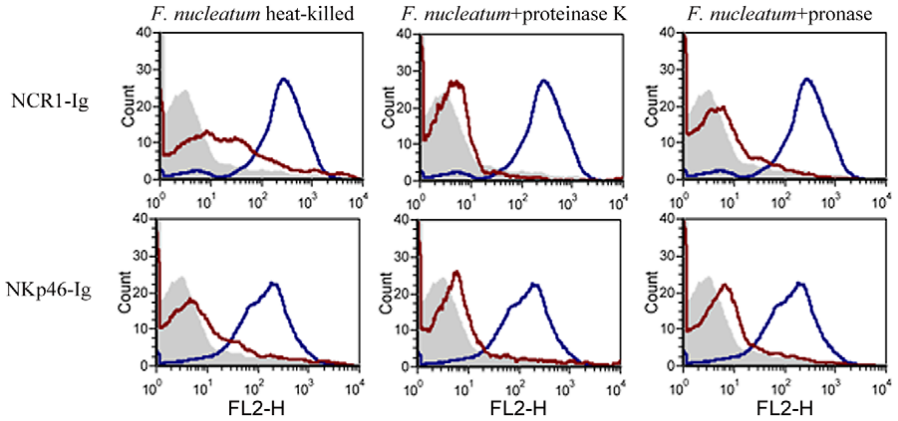
The *F. nucleatum* ligand is sensitive to heat, proteinase K and pronase treatment. Viable untreated, or treated (treatments are indicated above the histograms) *F. nucleatum* bacteria were stained with 5 µg of NCR1-Ig (upper) and of NKp46-Ig (lower). Filled grey histograms represent the staining with the NKp46D1-Ig. The blue histograms represent the staining of the untreated *F. nucleatum* and the red histograms represent the staining of the treated *F. nucleatum*. One representative experiment out of three is shown.

Figure 8 shows that the *F. nucleatum* ligand is either a protein or a modification found on a protein, as heat, proteinase K and pronase treatments significantly reduced the binding of both the NCR1-Ig and the NKp46-Ig fusion proteins.

### The direct interaction between *F. nucleatum* and NCR1 is functional, resulting in TNF-α secretion

**Figure 9:**
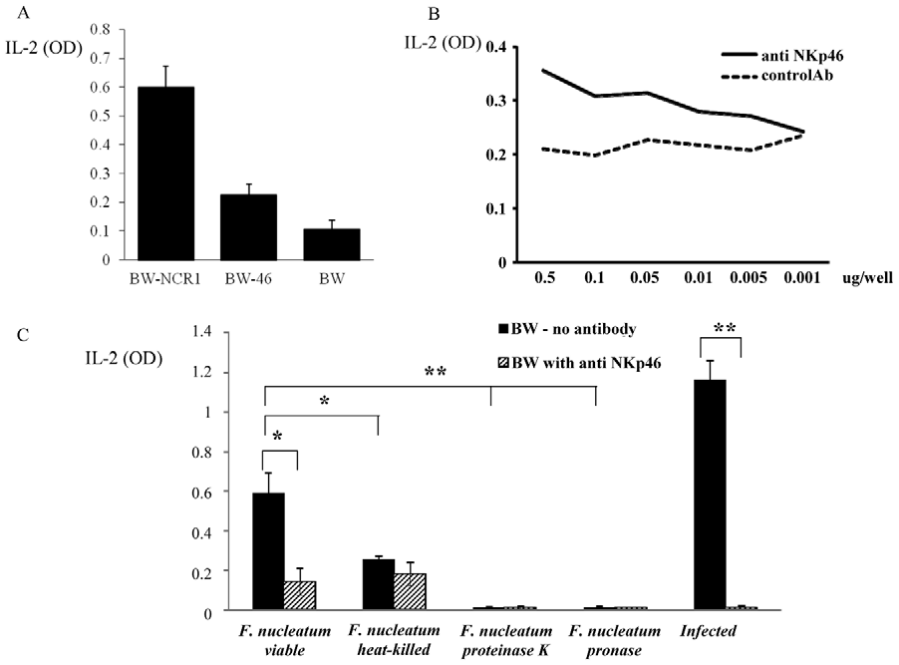
Functional reporter assays. (A) Basal IL-2 secretion of BW-NCR1, BW-NKp46 and parental BW cells. (B) BW-NKp46 transfected cells were cultured for 48 h in presence of plate bound (dosages indicated in the X axis) anti NKp46 mAb and control mAb. (C) 5×10^4^ BW-NKp46 transfected cells were cultured for 48 hours, with treated or untreated *F. nucleatum* and with 721.221 cells infected with PR8 influenza virus in the presence and in the absence of anti-NKp46 mAb. IL-2 secretion was determined by ELISA. Data represent absorbance at 650 nm ± SD and are average of two different experiments * *p*<0.05, ** *p*<0.01.

Next, we tested whether the direct interaction of *F. nucleatum* and NCR1 is functional. For this purpose, we initially used a reporter system in which we fused the NCR1 receptor to mouse CD3ζ chain and expressed this chimeric receptor in mouse BW cells. Triggering of this chimeric receptor will lead to the secretion of mouse IL-2, thus reporting for functional interaction of NCR1 with its ligand. However, upon generating this reporter system, a substantial secretion of IL-2 was observed, even without the addition of the bacteria (Figure 9A). A possible explanation to this observation is that the mouse BW cells express an unknown tumor ligand for the mouse NCR1 which consistently triggers IL-2 secretion as BW cells are specifically recognized by NCR1-Ig (data not shown).

Because we demonstrated above that both the mouse NCR1 and the human NKp46 receptors directly interact with *F. nucleatum*, we next examined whether we could use the human NKp46 protein fused to zeta as our reporter system. Importantly, as can be seen in Figure 9A, the self-secretion of IL-2 from the BW-46 cells was much lower than that of BW-NCR1. Furthermore, the NKp46 reporter system was functional as triggering of NKp46-zeta on BW cells by using plate bound anti-NKp46 resulted in a dose dependent IL-2 secretion (Figure 9B).

Therefore, BW-46-zeta transfectants were incubated with viable *F. nucleatum*, with heat-killed *F. nucleatum* and with *F. nucleatum* treated with proteinase K or with pronase. PR8 influenza virus infected 721.221 cells were used as positive control (Figure 9C). Incubations were performed for 48 hours in the presence, or in the absence of anti-NKp46 mAb and IL-2 levels were measured in the supernatants. Figure 9C reveals that *F. nucleatum* induced a significant secretion of IL-2 from BW-NKp46 cells and this secretion was almost completely blocked by the anti-NKp46 mAb. Furthermore, (in agreement with the above binding results), heat-killed bacteria, or bacteria treated with proteinase K or with pronase induced little or no IL-2 secretion. The activation of this reporter system by hemagglutinin seems to be more efficient as compared with *F. nucleatum* as incubation of the BW-NKp46 cells with infected 721.221 cells resulted in pronounced IL-2 secretion.

**Figure 10:**
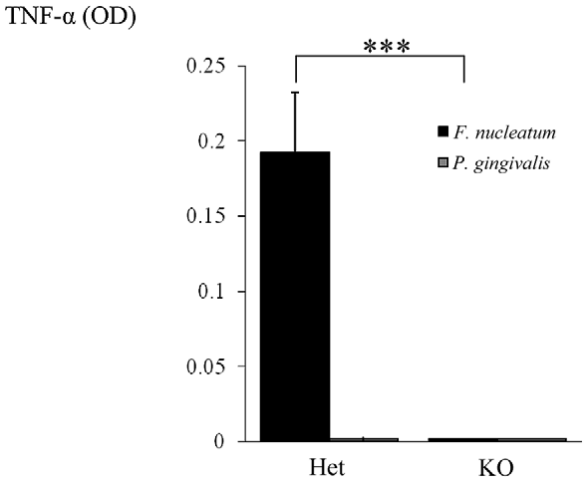
NCR1-dependent secretion of TNF-α following interaction with *F. nucleatum*. NK cells isolated from Ncr1*^+/gfp^* (Het) and from Ncr1*^gfp/gfp^* (KO) mice were cultured with *F. nucleatum* and with *P. gingivalis* bacteria for 48 hours. TNF-α secretion was determined by ELISA. Data represent absorbance at 650 nm ± SD and are average of two different experiments. *** *p*<0.001.

Our final effort was to demonstrate that the direct interaction between NCR1 and *F. nucleatum* will lead to TNF-α secretion. For this purpose we isolated (using the autoMACS instrument) mouse NK cells from Ncr1*^gfp/gfp^* and Ncr1*^+/gfp^* mice, incubated them with *F. nucleatum* or with *P. gingivalis* and tested the supernatants for the presence of TNF-α. As shown in Figure 10, no TNF-α secretion was observed when NK derived from the Ncr1*^gfp/gfp^* mice were incubated with *F. nucleatum*, while a significant secretion was detected when the Ncr1*^+/gfp^* mice were used. The TNF-α secretion was *F. nucleatum*-specific since *P. gingivalis* had no effect (Figure 10).

## Discussion

Periodontitis is a bacterial-induced inflammatory process that leads to the destruction of the tooth-supporting tissues and to tooth loss. It is one of the most common chronic inflammatory diseases in humans [44] and has been suggested as a risk factor for variable systemic conditions including rheumatoid arthritis, atherosclerosis and preterm births [45]–[47].

Here we investigated whether NK cells are involved in periodontitis through the interaction between their specific NK receptor NKp46 with periodontal bacteria. We used two complementary animal models. In the first model we induced experimental periodontitis by orally infecting the Ncr1*^+/+^* and Ncr1*^gfp/gfp^* animals with periodontal pathogenic bacteria. We show that in the absence of NCR1, when mice are infected with *F. nucleatum*, no loss of alveolar bone around the teeth is observed, while when mice were infected with *P. gingivalis* bone loss was observed independent of NCR1.

Due to technical and ethical limitations additional experiments could not be investigated in this model and we therefore used the subcutaneous chamber model [40], [41] to study the NKp46/NCR1 activity with regard to periodontal pathogens. Interestingly, we noticed that a large fraction of immune cells present in the inflamed chambers express NK cell markers such as NKG2D, DX5 and NK1.1, however these cells were not NK (GFP−), or T cells (CD3−). The identity and the function of these cells will be elucidated in future studies.

We show that in response to challenge with *F. nucleatum* and *P. gingivalis*, NK cells accumulate in the inflamed chambers, that *F. nucleatum* trigger the secretion of TNF-α and that the presence of TNF-α in the chambers is NCR1-dependent.

The interactions of NK cells with the bacteria probably renders them sensitive to *in vitro* manipulations and therefore we were not able to directly demonstrate that, in the chambers, NK cells are the main producers of TNF-α. Nevertheless, it is likely that TNF-α is indeed secreted by NK cells, *in vivo*, in response to *F. nucleatum* because upon incubation of isolated NK cells with *F. nucleatum*, an NCR1-dependent TNF-α secretion is observed.

Because alveolar bone loss is observed following *P. gingivalis* infection and since TNF-α could hardly be detected in the chambers following *P. gingivalis* infection we suggest that TNF-α has little role in the induction of the *P. gingivalis*-mediated bone loss in the system we used.

TNF-α is a very potent proinflammatory cytokine having pleotropic effects on both the immune and the skeletal systems. Its role and its contribution to the pathogenesis of chronic periodontitis, tissue destruction and bone loss has been clearly documented [48]–[50] and administration of anti-TNF-α antibodies resulted in reduced alveolar bone loss [51]–[54].

Previous studies demonstrated that the direct recognition of tumor cells [21], [43], viral infected cells [17], [43] and beta cells [20] by NKp46 (NCR1) lead to killing and to the secretion of TNF-α and IFN-γ [22], [55]–[57]. In our experimental system TNF-α, but not IFN-γ was present in the chambers. Similar observations were noted in other studies and it was proposed that NK cells shape their functional phenotype depending on the nature of the stimuli. For example, some tumors were able to induce TNF-α, but not IFN-γ secretion when co-cultured with naïve NK cells and only when NK cells were activated with IL-2, a significant increase in IFN-γ could be seen [58]. Another study showed that IL-4 compromised the ability of stimulated NK cells to release IFN-γ, without affecting the secretion of TNF-α [15] and interestingly IL-4 was detected in the chambers' exudates (data not shown).

The TNF-α response to *F. nucleatum* was NCR1-dependant, however, in the chamber experiments, in the absence of NCR1, the TNF-α secretion was markedly reduced but not totally abrogated. In contrast, no TNF-α secretion was observed from purified NK cells lacking NCR1 that interacted with *F. nucleatum*. This suggests that in the chambers, cells other than NK cells probably provide additional source for TNF-α. Indeed, previous studies showed that *F. nucleatum*-derived lipopolysaccharide up-regulated the secretion of the TNF-α by macrophage-like cells [59]. As shown here, macrophages and DCs are also present in the *F. nucleatum*-inflamed chambers and therefore we can speculate that the low level of TNF-α observed in the chambers implanted in the NCR1*^gfp/gfp^* animals is released either by macrophages recruited into the chambers, or by other immune cells.

Twenty four hours following bacteria inoculation, the TNF-α levels dramatically dropped to almost baseline levels, in spite of the continuous accumulation of NK cells in the chambers. This transient nature of TNF-α response has been reported previously [60], [61] and we assume that the TNF-α levels decline due to the elimination of *F. nucleatum* (the stimulating antigen) from the chambers during the study period.

In summary, we provide here the first evidence that NKp46 and NCR1 directly recognize a periodontal pathogen and we show that this recognition is heat, proteinase K and pronase sensitive, suggesting that the bacterial ligand is a protein or modifications of protein. Our data support the idea that periodontal pathogens initiate the disease by activating host mechanisms that consequently contribute to the destruction of the supporting apparatus of the periodontium by releasing inflammatory mediators, such as TNF-α.

## Materials and Methods

### Mice

The generation of the Ncr1*^gfp/gfp^* knockout mice has been previously described [21]. All experiments were performed in the specific pathogen–free unit of Hadassah Medical School (Ein-Kerem, Jerusalem) according to guidelines of the ethical committee. Ethics statement: This study was carried out in strict accordance with the Law for the Prevention of Cruelty to Animals (1994) and the regulations of the Hebrew University of Jerusalem Ethics Committee for Maintenance and Experimentation on Laboratory Animals. The protocol was approved by the Committee on the Ethics of Animal Experiments of the Hebrew University of Jerusalem (Permit Number: 11-5413). All surgery was performed under sodium pentobarbital anesthesia, and all efforts were made to minimize suffering.

### Bacterial strains and growth conditions

*P. gingivalis* strain ATCC3327 and *F. nucleatum* strain PK1594 were grown in an Bactron II anaerobic (N_2_∶CO_2_∶H_2_, 90∶5∶5) chamber (Sheldon Manufacturing Inc., Cornelius, OR) at 37°C in Wilkins-Chalgran anaerobic broth (Fluka, Spain) anaerobic chamber. *E. coli* strain ATCC25922 was grown in LB broth (Lennox, BD, Maryland, USA). Bacterial purity was determined by phase contrast microscopy. Bacteria were harvested during the logarithmic phase, washed by centrifugation and resuspended in phosphate-buffered saline (PBS) at 10^7^ bacteria/ml [40], [62]. Heat-killing was performed at 100°C for 10 min.

### Experimental periodontitis model

Infection was carried out as described by Baker at al. 2000 and Wilensky et al. 2009 [63], [64]. In brief, four to five-week-old Ncr1*^+/+^* (WT) and Ncr1*^gfp/gfp^* (KO) mice were given sulfamethoxazole/trimethoprim 0.08% and 0.016% respectively, in drinking water, *ad libitum* for 10 days. Three days following the withdrawal of antibiotics (day 14), the animals were infected with *F. nucleatum* or with *P. gingivalis* (2×10^8^ CFU in 0.2 ml of PBS and 2% carboxymethylcellulose) or vehicle only. The infection was carried out by gavage into the esophagus and oral cavity 3 times, once every other day. Forty two days after the last infection, the mice were sacrificed and the hemi-maxillae were collected and prepared for bone loss measurements using the micro computerized-tomography (μCT) technique [38]. At the end of the experiments mice were killed by CO_2_.

### Quantification of alveolar bone loss

For quantitative 3-dimensional analysis of the alveolar bone loss, the hemi-maxillae were examined by a desktop micro-CT system (μCT 40, Scanco Medical AG, Bassersdorf, Switzerland). The sagittal plan of the specimens was set parallel to the X-ray beam axis. The specimens were scanned at a resolution of 12 µm in all 3 spatial dimensions. The scans were Gaussian-filtered and segmented using a multi-level global thresholding procedure for the segmentation of enamel, dentin and bone. Residual supportive bone volume (RSBV) was determined separately for either root (bucco-mesial and bucco-distal) using a direct 3-dimensional approach [65]. The measured mesio-distal length of the alveolar bone was 204 µm and 120 µm for the mesio-buccal and the disto-buccal roots, respectively. The apical basis of the measured volume was set mesio-distally parallel to the cemento-enamel junction (CEJ) and bucco-palatinally parallel to the occlusal plane. The results represented the residual bone above the reference plane in mm^3^ [38].

### Subcutaneous chamber model

Two titanium coil chambers were inserted subcutaneously into anesthetized 6–8 week-old male mice as previously described [41]. After 10–14 days, the chambers were thoroughly emptied of their exudates and *P. gingivalis*, *F. nucleatum* and *E. coli* (10^6^ bacteria in 100 µl of PBS) were immediately injected into each chamber. The exudates were collected at 2 and 24 h post-infection (each chamber was sampled only once). After centrifugation at 4000 rpm for 20 min, the supernatants were collected for bacterial counts and cytokine analysis. To remove red blood cells the pellets were re-suspended in 3 ml of cold lysis buffer containing NH_4_Cl (1.55M), KHCO_3_ (0.138M) and NaEDTA (0.684 mM) at pH 7.3 for 3 min, after which the cells were washed and re-suspended in 0.5% BSA/PBS for flow cytometry analysis.

### Flow cytometry

Antibodies specific for NK cells (DX5; Caltag Laboratories, Burlingame, CA, USA), NKG2D (CX5; eBioscience, San Diego, CA, USA) were conjugated to phycoerythrin. DC were identified by CD11c (eBioscience, San Diego, CA, USA), macrophage with F4/80 (eBioscience, San Diego, CA, USA) and T cells were stained with CD3, CD4 and CD8 (BD Pharmingen, San Diego, CA, USA). NK1.1 antibody was produced in PK136 hybridoma cell line, as previously described [66]. Staining of NK1.1 was visualized with a secondary Cy5-conjugated strepavidin (Jackson ImmunoResearch Laboratories, West Grove, PA, USA). For intracellular staining cells were washed with Perm Wash (BD Pharmingen, San Diego, CA, USA), fixed and treated with Brefeldin A (Cell Signaling Technology) for 5 hours at 37°C. Cells were then incubated with antibodies to TNF-α (BD Pharmingen, San Diego, CA, USA).

### Cytokine analysis

The levels of TNF-α in chamber exudates were determined by ELISA as previously described [67].

### Determining the presence of viable bacteria in the chambers

Ten microliters of chamber exudates was serially diluted in duplicates in PBS and plated on tryptic soy agar containing sheep blood (Hylabs, Rehovot, IL). Plates with exudates from chambers challenged with *P. gingivalis* and *F. nucleatum* were grown under anaerobic conditions for 5–7 days at 37°C, while plates with exudates from *E. coli* challenged mice were grown in aerobic conditions at 37°C for 24 hours. The bacteria in the different colonies were identified by phase contrast microscopy. In addition, *P. gingivalis* colonies were confirmed by their black pigment.

### Staining of bacteria with soluble fusion proteins

The generation of NCR1-Ig, NKp46-Ig, NKp46D1-Ig, NKp44, NKG2D, NKp30 and CD16 was previously described [17], [18]. Proteins were purified on protein A/G columns as previously described [17], [18]. 2×10^5^ bacteria were stained with 5 µg fusion proteins (unless indicated otherwise). Staining was performed in the absence of azid and was visualized using a Phycoerythrin or Allophycocyanin conjugated anti-human Ig antibody.

### Interleukin-2 (IL-2) release assays from mouse lymphoma BW cells transfectants

Generation of the stable transfectants BW-NCR1 and BW-NKp46 was previously described [17]. 10^5^ treated and untreated *F. nucleatum* and 5×10^4^ influenza PR8-infected or uninfected 721.221 cells were incubated with 5×10^4^ BW-NKp46 cells in the presence and in the absence of 0.5 µg/well anti-NKp46 mAb (generated in our laboratory). All assays were performed in medium that do not contain antibiotics, at 37°C for 48 hours. The presence of IL-2 was measured by ELISA kit (BD Pharmingen, San Diego, CA, USA).

### TNF-α release assays from freshly-isolated NK cells

NK cells were isolated from splenocytes obtained from Ncr1*^+/gfp^* and from the Ncr1*^gfp/gfp^* mice by using an NK isolation kit (Miltenyi Biotech, Auburn, CA).The percentage of GFP^+^ cells was assessed by FACS and 5×10^4^ NK cells were cultured with 10^5^ *F. nucleatum* and *P. gingivalis* bacteria for 48 hours at 37°C. The presence of TNF-α was determined by ELISA.

## Supporting Information

Figure S1 ***F. nucleatum*** **remain intact following heat, protease K, and pronase treatment.** Phase microscopy (×1,000 magnification) and q-PCR quantification of treated and untreated bacteria.
(TIF)
