# Supplementary figures and images for: Direct Recognition of Fusobacterium nucleatum by the NK Cell Natural Cytotoxicity Receptor NKp46 Aggravates Periodontal Disease
Source: PLoS Pathog. 2012 Mar 22;8(3):e1002601. doi: 10.1371/journal.ppat.1002601 (PMC3310798; doi:10.1371/journal.ppat.1002601)

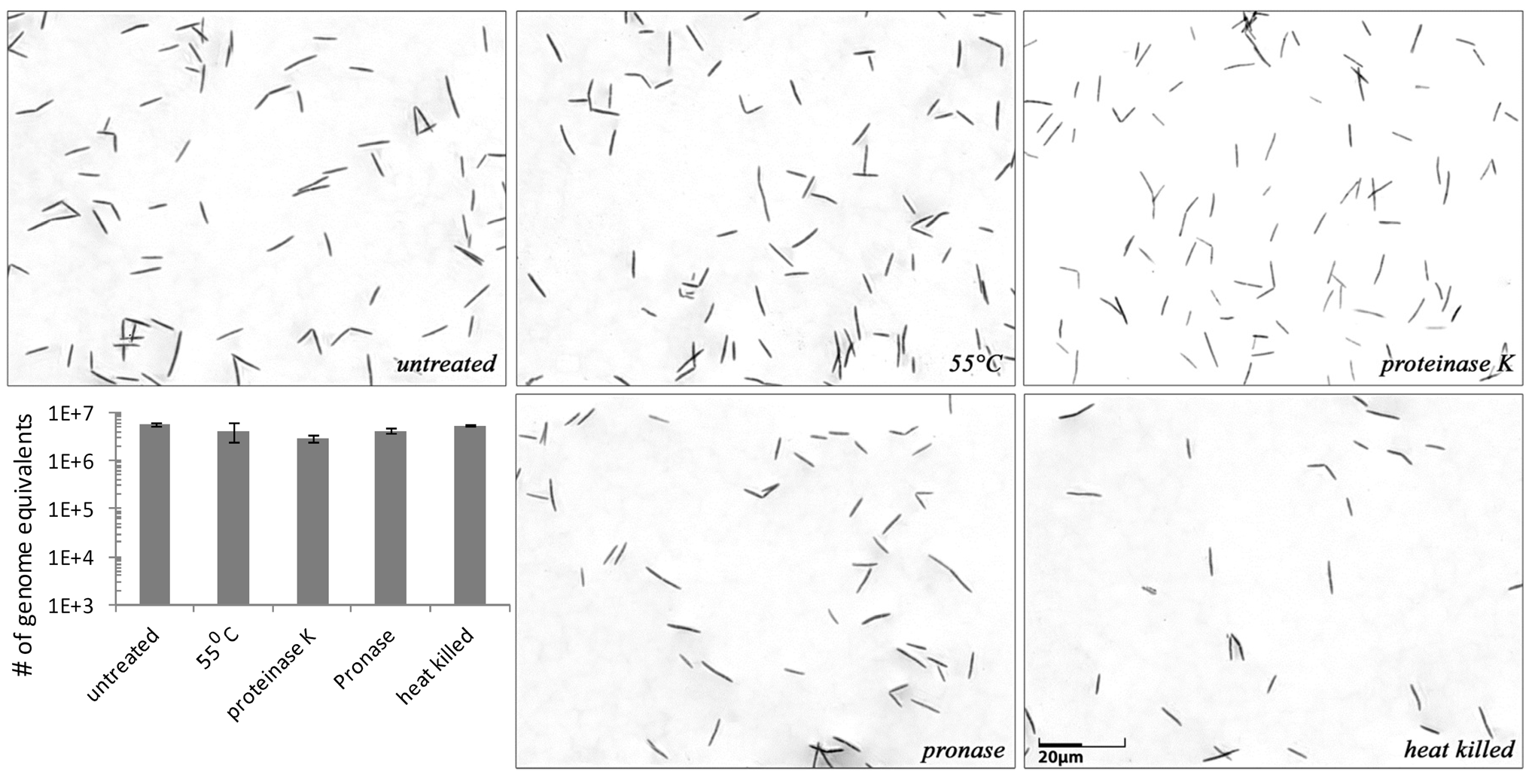

Supplement: Figure S1 — F. nucleatum remain intact following heat, protease K, and pronase treatment. Phase microscopy (×1,000 magnification) and q-PCR quantification of treated and untreated bacteria. (TIF) [file ppat.1002601.s001.tif]
